# Detecting variable (V), diversity (D) and joining (J) gene segment recombination using a two-colour fluorescence system

**DOI:** 10.1186/1759-8753-1-9

**Published:** 2010-03-01

**Authors:** Gina B Scott, Erika A de Wynter, Graham P Cook

**Affiliations:** 1 Leeds Institute of Molecular Medicine, University of Leeds, Leeds, UK

## Abstract

**Background:**

Diversity of immunoglobulins and the T cell antigen receptors is achieved via the recombination activating gene (RAG)-mediated rearrangement of variable (V), diversity (D) and joining (J) gene segments, and this underpins the efficient recognition of a seemingly limitless array of antigens. Analysis of V(D)J recombination activity is typically performed using extrachromosomal recombination substrates that are recovered from transfected cells and selected using bacterial transformation. We have developed a two-colour fluorescence-based system that simplifies detection of both deletion and inversion joining events mediated by RAG proteins.

**Results:**

This system employs two fluorescent reporter genes that differentially mark unrearranged substrates and those that have undergone RAG-mediated deletion or inversion events. The recombination products bear the hallmarks of true V(D)J recombination and activity can be detected using fluorescence microscopy or flow cytometry. Recombination events can be detected without the need for cytotoxic selection of recombination products and the system allows analysis of recombination activity using substrates integrated into the genome.

**Conclusions:**

This system will be useful in the analysis and exploitation of the V(D)J recombination machinery and suggests that similar approaches could be used to replace expression of one gene with another during lymphocyte development.

## Background

**Figure 1:**
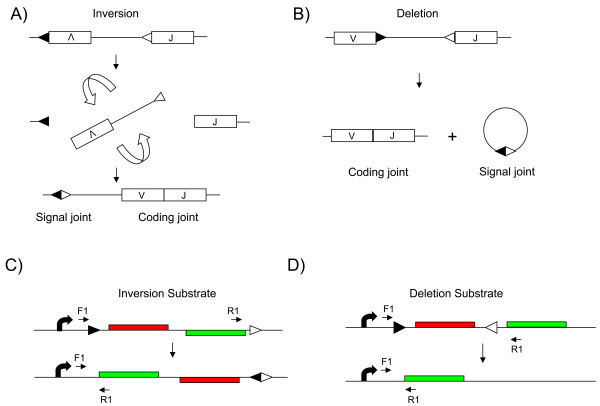
**Physiological variable (V), diversity (D) and joining (J) recombination and analogous recombination substrates**. (a) V and J segments on opposite strands (as found in the human Igκ locus) are joined by inversion between the recombination signal sequence (RSS)12 (filled triangle) and RSS23 (open triangle) motifs to generate a linked signal joint and coding joint (the VJ rearrangement). (b) V and J segments located on the same strand (as found in the human Igκ and λ loci) are recombined by deletion of the intervening DNA, leaving the coding segment on the chromosome and the signal joint on an excised circle of DNA. (c) In the inversion substrate, the DsRed gene and the EGFP gene are located on opposite stands, flanked by RSS12 and RSS23 motifs. V(D)J recombinase activity flips the segment allowing DsRed to be replaced by EGFP. (d) In the deletion substrate, the RSS motifs are in opposite orientations and flank the DsRed gene. On recombination, the DsRed gene is deleted, placing EGFP adjacent to the promoter. A single promoter is present in both constructs (curved arrow). The positions of the primer sequences F1 and R1, which were used to analyse recombination at the DNA level and for RT-PCR analysis, are shown.

The antigen receptor loci of B and T lymphocytes exhibit a unique mechanism of control amongst the genes of multicellular organisms. The production of functional immunoglobulin (Ig) and T cell receptor (TCR) genes is accomplished through a tightly regulated process of recombination. Variable (V), diversity (D) and joining (J) gene segments of antigen receptor loci are assembled into a functional coding unit by a series of site-specific recombination events mediated by the products of recombination activating gene (RAG)1 and RAG2 [1]. Recombination is targeted to specific sites by the recombination signal sequences (RSS), which flank the gene segments. RSS motifs consist of a conserved heptamer (CACAGTG) separated from a conserved nonamer (ACAAAAACC) by a spacer of variable sequence of either 12 or 23 base pairs (bp). Recombination occurs between an RSS with a 12-bp spacer (RSS12) and an RSS with a 23-bp spacer (RSS23) and the intervening DNA is either deleted or inverted depending upon the orientation of the two signals (Figure 1). Double strand breaks introduced at the RSS motifs by the RAG proteins are then resolved by non-homologous end joining. Two products are generated, a signal joint in which the RSS motifs are joined and a coding joint (Figure 1) in which the gene segments are joined [2].

Assays of V(D)J recombination have relied extensively upon the transfection of extrachromosomal plasmid substrates into RAG-expressing cell lines and the recovery of these plasmids in *Escherichia coli *[3-6]. Many of these substrates are designed such that V(D)J recombination allows expression of a selectable marker in bacteria [3-5]. This approach has been extremely valuable in dissecting the basic mechanisms of recombination. However, since these substrates are extrachromosomal, this approach cannot be used to analyse the effect of chromatin structure on the recombination process. Alternatives have been described which rely on the recombination of integrated genes encoding selectable markers [7-9], or recombination to generate a single fluorescent gene product [10-12]. Furthermore, some of these techniques have been applied in studies of recombination in transgenic mice [10]. Here, we describe a system whereby V(D)J recombination substrates are stably integrated into the host cell genome and both non-recombined and recombined products can be detected by fluorescence.

## Results and Discussion

V(D)J recombination can occur either by deletion of the DNA between RSS motifs or by inversion of the intervening segment (Figure 1a, b). We generated vectors to assess both types of recombination. The system utilises two fluorescent proteins; DsRed, derived from the coral *Discosoma *and enhanced green fluorescent protein (EGFP). The system was designed such that unrearranged substrates would express the DsRed gene, whereas substrates that had undergone RAG-mediated recombination would replace DsRed expression with EGFP expression. In one construct, the EGFP open reading frame was placed on the opposite strand to DsRed and the RSS motifs arranged such that the segment containing DsRed and EGFP would be inverted by RAG-mediated recombination (inversion substrate; Figure 1c). In the other construct, the two reporter genes were in the same orientation, with the DsRed gene flanked by RSS12 and RSS23 motifs, allowing deletion of the DsRed gene by RAG-mediated recombination (deletion substrate; Figure 1d).

**Figure 2:**
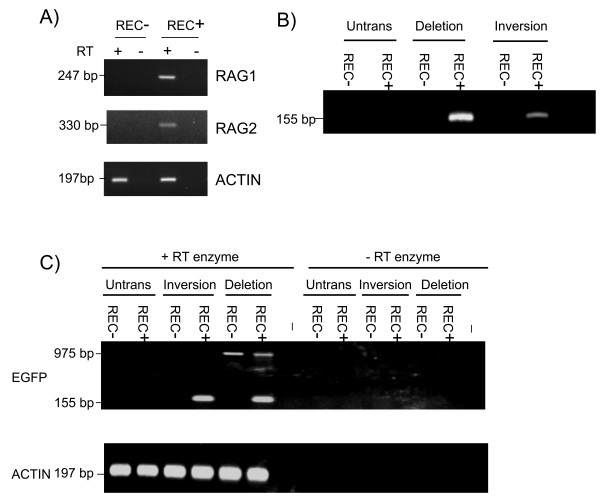
**Cell-type specificity of recombination**. (a) Recombination activating gene (RAG)1 and RAG2 expression in pre-B 300-19P cells (REC+) and WEHI231 B cells (REC-) analysed by reverse transcription polymerase chain reaction (RT-PCR). Actin transcripts were detected in both cell lines. The RAG genes are intronless and assays were performed using reverse transcriptase (RT+) as well as in its absence (RT-) to ensure that signals were not derived from contaminating genomic DNA. (b) Detection of recombination by PCR (using the F1 and R1 primers shown in Figure 1). A PCR product of 155 bp was predicted following recombination. (c) RT-PCR to detect expression of EGFP mRNA from the transfected substrates. The PCR product diagnostic of EGFP transcripts from the recombined substrate is 155 bp. The 975-bp product detected from the deletion substrate transcripts represents a mRNA that traverses the entire DsRed gene and continues on through the EGFP gene. No products were detected in the absence of reverse transcriptase (- RT enzyme). Both DNA recombination and mRNA expression assays were performed from untransfected cells (Untrans) and in cells stably transfected with the deletion and inversion substrates (non-clonal populations grown under continuous selection) using 300-19P (REC+) and WEHI231 (REC-) cells. A (-) symbol indicates no added template.

The inversion and deletion constructs were stably transfected into two cell lines representing different developmental stages of the B cell lineage. The cell line 300-19P is a pre-B cell line, expresses RAG1 and RAG2 and is known to catalyse V(D)J recombination, whereas WEHI231 is a B cell line in which RAG gene expression has been silenced and is therefore unable perform V(D)J recombination. The expression of RAG1 and RAG2 in these two cell lines was confirmed using reverse transcription polymerase chain reaction (RT-PCR) (Figure 2a). Pools of stable transfectants were analysed for recombination products at the DNA level using PCR. The results shown in Figure 2b indicated that recombination of both deletion and inversion constructs was restricted to the RAG-expressing 300-19P cells.

These transfectants were then analysed for expression of the DsRed and EGFP reporter genes at the mRNA level. RT-PCR analysis established that expression of the EGFP gene was restricted to the RAG-expressing cell line and that EGFP transcripts could be detected in cells transfected with both the inversion and deletion construct (Figure 2c). A larger RT-PCR product resulting from the transcription through the DsRed open reading frame and continuing into EGFP was detected in cells transfected with the deletion substrate (Figure 2c).

**Figure 3:**
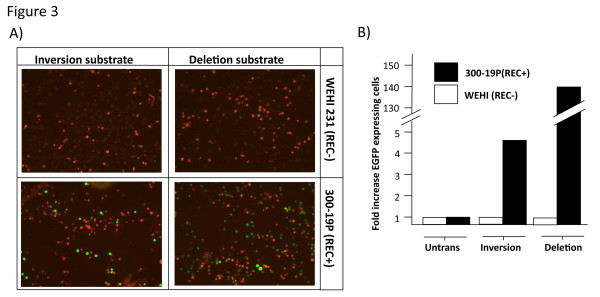
**Expression of the recombined substrates**. (a) Fluorescence microscopy of EGFP and DsRed expression. The four panels show expression of the DsRed (from unrearranged template) and rearranged EGFP gene products from inversion and deletion substrates in both 300-19P and WEHI231 cells. (b) Flow cytometric analysis of 4-week cultures of untransfected and transfected recombinant 300-19P (REC+) and WEHI231 (REC-) cell lines. The fold increase in number of EGFP-expressing cells was established by assigning an arbitrary value of 1 for untransfected cells.

Our aim was to identify V(D)J recombination at the single cell level. We therefore analysed expression of the DsRed and EGFP molecules by fluorescence. Fluorescence microscopy of the stably transfected RAG negative WEHI231 and RAG positive 300-19P cells indicated that expression of EGFP was restricted to the 300-19P cells. Furthermore, EGFP expression was clearly detectable in both the deletion and inversion transfectants (Figure 3a). Expression was further analysed by flow cytometry (Figure 3b). Untransfected WEHI231 and 300-19P cells were assigned an arbitrary fluorescence level of 1 unit and compared to the fluorescence observed following stable transfection with the deletion and inversion constructs. Expression of EGFP was detectable by flow cytometry in both deletion and inversion transfectants with the number of EGFP-expressing cells greater in the deletion substrate transfected population than the inversion substrate population. The PCR-based detection of DNA recombination shown in Figure 2b was not quantitative. However, these PCR results also suggest a lower level of recombination of the inversion construct compared to the deletion construct, a conclusion that has previously been reached using extrachromosomal substrates [3]. However, inversion events are a normal process in the assembly of antigen receptor loci and can occur over megabase-length regions of the chromosome [13].

The process of V(D)J recombination has evolved to generate a repertoire of antigen receptors. Large repertoires are generated by both combinatorial diversity and by the imprecise nature of the recombination process whereby the location of the double strand break is not fixed and nucleotides can be added or deleted from the free DNA end, generating extensive diversity in complementarity determining region (CDR)3 of the antigen receptor [14-16]. Cloning and sequencing of the coding joints from both deletion and inversion substrates transfected into 300-19P cells revealed that a number of independent recombination events had occurred in the transfected cell population and that recombination of both constructs bore the typical hallmarks of V(D)J recombination, namely the introduction of diversity at the recombination junction via insertion and deletion of sequences (Table 1).

**Table 1:** Diversity at coding joint sequences

RSS23 coding end sequence	Nucleotides lost	Nucleotides added	Nucleotides lost	RSS12 coding end sequence
ATTACGCGC*				*GGTACCGTC
Deletion substrate:				
ATTACGCG	-C	+GC	-G	GTACCGTC
ATTACG	-CGC		-GG	TACCGTC
ATTACGC	-GC		-GGTAC	CGTC
ATTACGCG	-C	+GG	-GGTA	CCGTC
ATTACGCG	-C		-GG	TACCGTC
ATTACGCG	-C	+GCG	-GGTA	CCGTC
ATTACG	-CGC		-G	GTACCGTC
ATTACGCG	-C		-G	GTACCGTC
ATTACGCG	-C		-G	GTACCGTC
Inversion substrate:				
ATTACGCGC	-C	+G	-GGTA	CCGTC
ATTACG	-CGC		-GG	TACCGCG
ATTACGCG	-C	+GC	-GGT	ACCGTC
ATTACGCG	-C			GGTACCGTC
ATTACGCG	-C	+GC	-GGT	ACCGTC
ATTACGCG	-C	+GC	-GGT	ACCGTC
ATTACG	-CGC		-GG	TACCGTC
ATTACGC	-GC		-GGTAC	CGTC
ATTACGCG	-C	+GC	-G	GTACCGTC
ATTACGCG	-C		-G	GTACCGTC

DNA is cleaved at the boundary of the recombination signal sequence (RSS) motifs and the ends are joined. Joining involves the trimming of nucleotides and the addition of bases via terminal deoxynucleotide transferase (TdT), resulting in diversity at the coding joint.

*The sequences marked indicate the predicted sequence if recombination involved no nucleotide loss or addition. The actual sequences obtained for the deletion and inversion substrate are shown, with nucleotides loss and gained indicated.

In the system described here, the use of the twin fluorescent protein system distinguishes individual cells that have recombined a V(D)J recombination substrate from those which carry the substrate in a configuration representing germline gene segments. The ability to assay the absence of recombination is important when testing the effects of mutations or local chromatin environment on the recombination process. Importantly, the twin fluorescent protein system allows recombination to be detected *in situ *in the absence of selection. Previously, assays of V(D)J activity have relied heavily upon extrachromosomal recombination substrates that are transiently transfected into cells before recovery and transformation into *E. coli *[3-6]. Chromatin structure is important in regulating V(D)J rearrangement [17,18] and future studies will benefit from the use of substrates, such as those describe here, that integrate into the chromosome and allow simple assessment of germline or rearranged configuration.

Interestingly, the twin fluorescent protein system also indicates that V(D)J recombination could be used to control the expression of exogenous genes. Just 67 bp of *cis*-acting sequences (RSS12 + RSS23) need to be provided to ensure correct expression of the exogenous gene (unpublished results). The ability to switch gene expression from one gene to another by harnessing endogenous recombinase activity may have practical applications in the B and T cell lineage analogous to the use of the Cre/Lox system or other exogenous recombinases [19].

## Methods

### Construction of recombination substrates

The inversion and deletion plasmids (shown in Figure 2) were constructed using pJMA2EGFP [20] and pDsRed-N1 (Clontech-Takara Bio Europe, Saint-Germain-en-Laye, France). Briefly, a double stranded oligonucleotide containing RSS12 was inserted upstream of DsRed, between the promoter and coding sequence. The EGFP sequence from pJMA2EGFP was then inserted downstream of DsRed, either on the same strand as DsRed (for the deletion construct) or on the opposite strand (for the inversion construct). An RSS23 motif was then added (using a double stranded oligonucleotide) either downstream of EGFP (for the inversion plasmid) or between DsRed and EGFP (for the deletion plasmid). The sequences of the RSS12 and RSS23 motifs are shown in Table 2. The precise details of the plasmid construction, including oligonucleotide sequences used, are available on request from the authors.

**Table 2:** Recombination signal sequence (RSS) motifs

Signal	Sequence
RSS12	CACAGTGctacagactggaACAAAAACC
RSS23	CACAGTGgtagtactccactgtctggctgtACAAAAACC

RSS motifs are comprised of a conserved heptamer and conserved nonamer (shown in upper case) separated by either 12 or 23 base pairs (bp) of intervening DNA (lower case).

### Cell culture and transfection

Murine B cell lineage cell lines (300-19P and WEHI231) were maintained in RPMI medium supplemented with 10% foetal calf serum and 50 μM 2-mercaptoethanol. The recombination substrates were transfected into pre-B (300-19P) and B (WEHI231) cell lines by electroporation. Approximately 20 μg of linearised DNA were electroporated (350 V, 700 μF) into 10^7 ^cells in a volume of 0.8 ml of medium. Cells were then incubated for 16 h in a total volume of 10 ml of medium before the addition of G418 (2 mg/ml) for selection.

### Nucleic acid isolation and PCR

DNA and RNA were isolated from 10 ml aliquots of culture using Genelute kits (Sigma-Aldrich Ltd, Poole, UK) as per the manufacturers' protocols. The optical density was measured at 260 nm and the concentration of the nucleic acid calculated; 500 ng of genomic DNA was used as template in a PCR reaction. Recombination events were detected by PCR using appropriate primers (shown in Figure 1) for 35 cycles of 94°C (1 min), 58°C (1 min) and 72°C (1 min) with a final extension step of 10 min at 72°C. PCR products encompassing the coding joint region of the recombined substrates were cloned into pcDNA3 and sequenced. For RT-PCR, reverse transcriptase (Invitrogen, Paisley, UK) was used to synthesise the first strand cDNA from 5 μg of total RNA using random primers (alongside negative controls lacking enzyme). The first strand synthesis reaction was then diluted fivefold and used as a template for PCR reactions to detect mRNA transcribed from rearranged DNA substrates (primer sequences available on request from the authors).

For RT-PCR of mouse genes, the following primer sequences were used; RAG1F, CCCGATGAAATTCAACACCC; RAG1B, CTTGACTTCCCATCAGCATGG; RAG2F, CCTGTCCTACTGGAGTCTTTC; RAG2B, GGCCGTATCTGGGTTCAGGG; β-actin F, TGCGTGACATCAAAGAGAAG; β-actin B, GATGCCACAGGATTCCATA. Reactions with these primers were performed as described for recombination assays.

### Flow cytometry

Cells transfected with deletion or inversion substrates were analysed for the expression of the EGFP protein (resulting from recombination of the substrate) using the FL1 channel of a FACS Calibur flow cytometer (BD Biosciences, Oxford, UK). The mean fluorescence intensity of EGFP expression was compared between transfected and non-transfected cells and the data expressed as a fold increase in fluorescence with non-transfected cells assigned an arbitrary value of 1.

## Competing interests

The authors declare that they have no competing interests.

## Authors' contributions

GPC designed the overall strategy, GPC and GBS designed the constructs, GBS performed the experiments, EADW assisted with flow cytometry and GPC and GBS wrote the paper.
